# Biallelic loss of BCMA as a resistance mechanism to CAR T cell therapy in a patient with multiple myeloma

**DOI:** 10.1038/s41467-021-21177-5

**Published:** 2021-02-08

**Authors:** Mehmet Kemal Samur, Mariateresa Fulciniti, Anil Aktas Samur, Abdul Hamid Bazarbachi, Yu-Tzu Tai, Rao Prabhala, Alejandro Alonso, Adam S. Sperling, Timothy Campbell, Fabio Petrocca, Kristen Hege, Shari Kaiser, Hervé Avet Loiseau, Kenneth C. Anderson, Nikhil C. Munshi

**Affiliations:** 1 Department of Data Science, Dana Farber Cancer Institute, Boston, MA USA; 2 Department of Biostatistics, Harvard T. H. Chan School of Public Health Boston, Boston, MA USA; 3 Department of Medical Oncology, Dana Farber Cancer Institute, Harvard Medical School, Boston, MA USA; 4 Department of Internal Medicine, Jacobi Medical Center, Albert Einstein College of Medicine, New York, NY USA; 5 VA Boston Healthcare System, Boston, MA USA; 6 Bristol-Myers Squibb, San Francisco, CA USA; 7 Bluebird Bio, Cambridge, MA USA; 8 Bristol-Myers Squibb, Seattle, WA USA; 9 University Cancer Center of Toulouse Institut National de la Santé, Toulouse, France

**Keywords:** Cancer genomics, Cancer microenvironment, Cancer immunotherapy, Cancer therapeutic resistance, Myeloma

## Abstract

BCMA targeting chimeric antigen receptor (CAR) T cell therapy has shown deep and durable responses in multiple myeloma. However, relapse following therapy is frequently observed, and mechanisms of resistance remain ill-defined. Here, we perform single cell genomic characterization of longitudinal samples from a patient who relapsed after initial CAR T cell treatment with lack of response to retreatment. We report selection, following initial CAR T cell infusion, of a clone with biallelic loss of BCMA acquired by deletion of one allele and a mutation that creates an early stop codon on the second allele. This loss leads to lack of CAR T cell proliferation following the second infusion and is reflected by lack of soluble BCMA in patient serum. Our analysis suggests the need for careful detection of BCMA gene alterations in multiple myeloma cells from relapse following CAR T cell therapy.

## Abstract (web summary)

Relapse following BCMA targeted CAR T-cell therapy is frequently observed in patients with multiple myeloma (MM). Here, by single cell transcriptome profiling on serially collected bone marrow samples, the authors report biallelic loss of BCMA as the mechanism of resistance underlying both relapse and lack of response to a second CAR T infusion in a patient with MM.

## Introduction

Chimeric antigen receptor (CAR) T-cell therapy targeting B-cell maturation antigen (BCMA) has provided frequent, deep, and durable responses in relapsed, refractory multiple myeloma (MM), with initial Phase I/II studies reporting 73–100% overall response and 31–69% complete response^1–3^. However, progression-free survival in some studies have been <12 months, indicating myeloma recurrence despite the persistence of CAR T cells in a number of cases^1,2^. Importantly, among the small number of patients retreated with the same CAR T cell product at the time of progression, responses have been infrequent^4,5^. This highlights development of acquired resistance mechanisms^6,7^, which may preclude effectiveness of the second CAR T infusion, and may also explain relapse following the initial CAR T-cell therapy.

In this work, by performing single-cell transcriptome profiling on serially collected bone marrow (BM) samples, we show biallelic loss of BCMA as one of the resistance mechanisms to anti-BCMA CAR T-cell therapy with Idecabtagene Vicleucel in a patient with initial response but relapse with resistance to retreatment with the same CAR T-cell product. Furthermore, our results also highlight that MM cells may develop alternative paths to survive without BCMA.

## Results

**Fig. 1:**
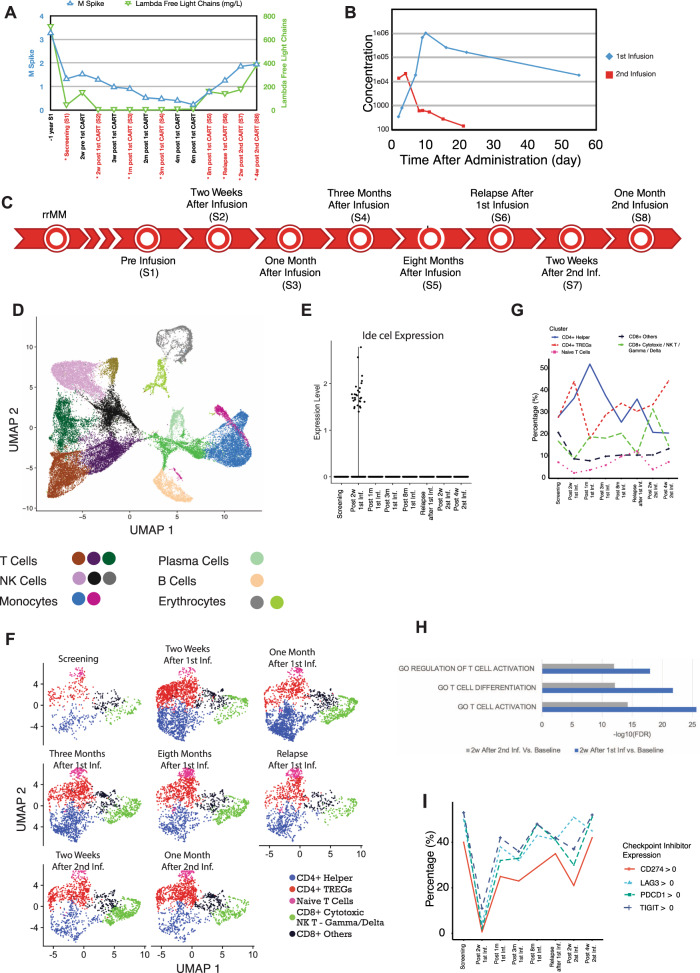
Response to CAR T cell treatment and microenvironment changes. **A** M spike and lambda free light chain evaluations for the patient. The *y* axis on left shows the M spike values (blue) and on the right it shows the lambda free light chain values (green). Time points (*x* axis) marked with red labels also shows the longitudinal sample collection for single-cell RNA sequencing. **B** Expansion of CAR T cells (*y* axis) measured with qPCR after first (blue) and second (red) infusions from day 0 to day 60 (*x* axis). **C** Timeline of the eight samples collected for single-cell RNA sequencing. **D** Thirteen single-cell clusters from eight longitudinal bone marrow samples. Annotation of cell clusters are marked in the bottom part with color codes. Cell embedings are shown by using UMAP1 and UMAP2. **E** Ide-cel expression in single cells. Only limited number of cells are CAR+ at 2 weeks after the first infusion. None of the other time points show CAR+ cells. **F** Re-clustered T cells divided by time points from study screening to 1 month after second infusion and T-cell annotations for CD4+ and CD8+ cells are shown with color codes. **G** Percentage of particular T-cell types (*y* axis) at each time point (*x* axis) evaluated with single-cell RNA seqeuecning for T-cell clusters (top figure legend). Percentages are reflecting the % of particular cluster at given time point within all T cell populations. **H** Gene-set enrichment FDR values for differentially expressed genes for two samples collected two weeks after first (blue) and second (green) infusions. **I** Percentage of T cells (*y* axis) expressing immune checkpoint inhibitors at each time point (*x* axis).

We evaluated samples from an individual patient who was diagnosed with IgG lambda MM with hypodiploidy and a complex karyotype with t(8;12) (q24;q14), clonal t(11;14) (q13;q32), and clonal deletion 13. The patient was treated with four lines of therapy including proteasome inhibitor, immunomodulatory agent, and anti-CD38 antibody before CAR T-cell therapy, with limited response. The patient was enrolled in a Phase I trial (CRB-401 ClinicalTrials.gov number, NCT02658929) of anti-BCMA CAR T-cell therapy with Idecabtagene Vicleucel (ide-cel) and received 150 × 10^6^ CAR+ T cells at day 0 following lymphodepletion with fludarabine (30 mg/m^2^ per day) and cyclophosphamide (300 mg/m^2^ per day) on days −5, −4, and −3, as reported in ref. ^1^. The patient developed grade 1 cytokine release syndrome and achieved partial response by 3 months. The patient relapsed 9 months after the first CAR T infusion and was treated a second time with identical lymphodepletion and using the same CAR T-cell product as the first infusion but at a higher dose of 450 × 10^6^ CAR+ T cells with no response (Fig. 1A, B).

### Changes in BM microenvironment post-CAR T-cell therapy

**Fig. 2:**
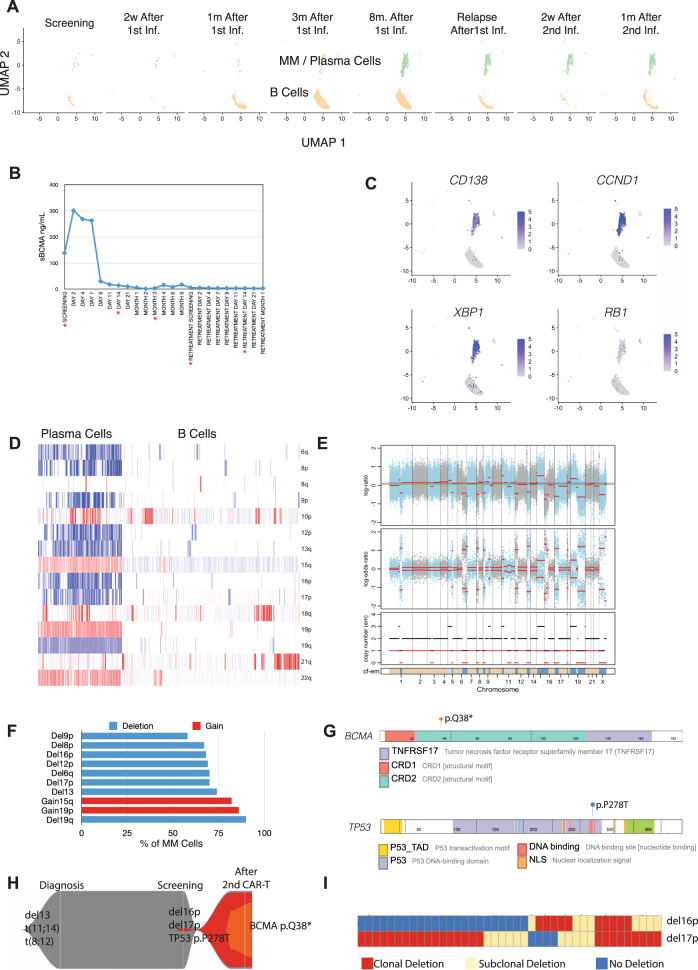
Tumor-intrinsic changes. **A** Cell embedings for plasma/multiple myeloma (MM) cells (green colors) and B cells (cream colors) are shown from screening to 2 weeks after second infusion (*x* axis). B cells are first detected at 1 month after first infusion and increased frequency until relapse. MM cells are detected at the 8 months after first infusion sample and remain same for further time points. **B** Soluble BCMA (sBCMA) level (*y* axis) at the study screening, after first infusion, relapse, and after second infusion (*x* axis) are shown. Time points makerd with * indicates the time points scRNAseq data also available. Screening refers to screening before first infusion (S1). Day 14 and Months 3 are refering to two weeks after first infusion (S2) and 3 months after first infusion (S4). Retreatment Screening is the relapse after first infusion (S6) and Retreatment Day 14 (S7) is 2 weeks after second infusion. **C** Expression levels of CD138, CCND1, XBP1, and RB1 in multiple myeloma (MM) cells and B cells. Normalized expression level scales are shown with legends. **D** Copy number predictions for each single cell (columns) from single-cell RNA sequencing data for chromosomal arms (rows). Deletions are shown with blue and gains are shown with red color for MM cells (left) and B cells (right). **E** Copy number estimates for CD138+ cells after second CAR T-cell infusion using whole exome sequencing. The top panel displays total copy number log-ratio where diploid state is shown with purple line and the second panel displays allele-specific log odds ratio data for allele-specific copy number calls with chromosomes alternating in blue and gray. Third panel shows the corresponding integer (black line for total copy number, red line for minor copy number) copy number calls. The bottom panel shows the predicted clonality of each events. Dark blue colors show regions with colonal copy number alterations and light blue color shows subclonal copy number events. **F** Percentage (*x* axis) of single multiple myeloma/plasma cells with various copy number deletions (del) or gains (*y* axis). Copy number events for each cell predicted using single-cell RNA sequencing. **G** Somatic mutaitons detected at relapse after second infusion with whole exome sequencing. Nonsense mutation which creates early stop codon in BCMA (top panel) and missense TP53 mutation (bottom panel) are shown in their amino acid locations. Protein domains are shown with color codes in each genes. **H** Clonal evolution of MM cells from diagnosis to relapse after second CAR T-cell infusion. **I** Co-occurrences of deletion 17p (del17p) and deletion 16p (del16p) on large-scale MM cohort. Clonal (red color), subclonal (yellow) deletions are shown for newly diagnosed MM patients (columns) and only patients with del16p and/or del17p are shown.

To delineate changes in BM cellular components as a potential mechanism underlying lack of response to CAR T-cell reinfusion, we performed single-cell transcriptome profiling on serially collected BM samples (Fig. 1C). Clustering analysis from 37,658 cells from 8 time points, before the first CAR T cell infusion to 1 month after the second infusion, identified 13 clusters consisting of hematopoietic cells and MM cells (Fig. 1D and Supplementary Fig. 1). The BM sample before the first infusion was depleted of CD138+ cells by cell selection. A small number of MM cells were observed at 2 weeks after the first infusion of the CAR T-cell therapy. Thereafter, MM cells became undetectable and remained undetectable until eight months after the first infusion, when biochemical as well as cytological relapse occurred (Fig. 2A and Supplementary Fig. 1B). We observed a predicted suppression of B-cell count at study entry as an effect of the MM cell growth, with B-cell recovery at 1 month coinciding with anti-MM response (3% of all cells) and reaching 18% at 8 months after first infusion (Supplementary Fig. 1B), and again suppressed to 3% at relapse. We detected CAR+ T cells in the BM only at 2 weeks after first infusion, when maximal CAR+ T-cell expansion was observed in blood using reverse-transcription PCR (RT-PCR)-based detection (Fig. 1B, E). We did not detect infused CAR T cells in the BM with single-cell transcriptome profiling after the second CAR T infusion, but a limited expansion was confirmed in the blood using RT-PCR (Fig. 1E). The 6-log expansion of CAR+ T cells in the blood after the first infusion is consistent with observed expansion in the KarMMA study, where 5.5 log expansion was observed in the responding patients with a median peak CAR+ T-cell expansion at day 11^2^. A lower expansion (2-log) with the second infusion may represent environmental influences or MM-intrinsic factors.

Re-clustering of the T-cell cluster showed an increased proportion of CD4+ helper and T-regulatory cells (Treg) 2 weeks after first infusion (Fig. 1F, G and Supplementary Fig. 2), and these two clusters had high expression of proliferation-related genes (Fig. 1H and Supplementary Data 1 and 2). However, Treg proportion remained similar at the second infusion, ruling out its impact on lack of expansion of CAR+ T cells. To detect any unusual endogenous T-cell activity that may potentially affect CAR T-cell function, we investigated inhibitory markers *CD274* (*PD-L1*), *PDCD1* (*PD-1*), *LAG3*, *TIGIT* at early and late time points including after second infusion. As can be seen (Fig. 1I and Supplementary Table 1), at no time point the proportion of the cells expressing these checkpoint inhibitors is higher then the base line. Moreover, the absence of change in these markers on endogenous T cells does not mean that the CAR+ T cells do not express these markers. However, in absence of detectable CAR T cells there is no direct way to look at expressed inhibitory markers on CAR T cells. Therefore, future studies will require investigation of resistance associated with presence of checkpoint inhibitors.

### Role of tumor intrinsic factors in resistance

As we did not delineate a role of the BM milieu mediating suppression of CAR T-cell expansion and function following second infusion, we next explored tumor intrinsic factors. We evaluated soluble BCMA (sBCMA) level (produced predominantly by MM cells) in serum at different time points, and observed high levels before the first CAR T cell infusion, which dropped significantly to a very low level coincident with the clinical response; however, sBCMA remained low even at the time of relapse with increased burden of MM, indicating a lack of BCMA production by MM cells (Fig. 2B). We therefore investigated genomic changes in MM cells at the time of relapse. This patient had clonal t(11;14) translocation (96% of all cells) and clonal deletion 13 (94% of all cells) at the time of diagnosis. A similar clonal composition was observed by fluorescence in situ hybridization (FISH) analysis at study enrollment, when 4% of the cells also showed deletion 17p. Our single-cell transcriptomic analysis of BM samples identified three samples (at the time of relapse and post second CAR T-cell infusion) with significant numbers of MM cells, evidenced by expression of CD138 and *XBP1* (markers of plasma cells), *CCND1* (upregulated in this patient with t(11;14)), and lack of *RB1* (downregulated in this patient with del13) (Fig. 2C). Imputation of copy number alterations from single-cell transcriptomic data showed that the majority of MM cells had a deletion of 16p, including the *BCMA* locus located on 16p13.13. (Fig. 2D). We further validated these findings using deep whole exome sequencing (WES) of purified CD138+ cells collected 2 weeks after the second CAR T infusion. Of note, copy number alterations detected by WES almost completely overlapped with CNAs predicted by single-cell RNA sequencing (scRNAseq), including deletion 16p (Fig. 2E). Before the first CAR T-cell infusion, 4% of BM MM cells showed deletion 17p, whereas after the second infusion both WES and scRNAseq prediction showed that del17p and del16p were clonal, and longitudinal scRNAseq analysis indicated that del17p and del16p co-occurred in the same clone (Fig. 2D, F). Interestingly, WES also identified a high subclonal (~70%) nonsense mutation (p.Q38*) in *BCMA* that creates an early stop codon in the *BCMA* gene (Fig. 2G and Supplementary Fig. 3). This biallelic *BCMA* loss, acquired with one copy deletion and a second copy loss-of-function mutation, provides the molecular basis for lack of *BCMA* expression in MM cells at the time of relapse.

## Discussion

This case represents initial response followed by development of an acquired resistant phenotype as represented by both relapse and then lack of response to second CAR T infusion (Fig. 2H). BCMA represents an important component of plasma cell function, and thus its loss is not frequently observed. However, this case highlights a possibility that myeloma cells may be able to acuire alternative growth mechanisms to survive without BCMA expression and related signaling intermediates. Studies have shown that MM usually shows substantial inter and intra-tumor heterogeneity, which is closely related to progression, resistance to therapy, and recurrences^8–12^. Loss of several other targets for different treatments, such as CRBN with immunomodulatory agents or BCL-2 with venetoclax, has been associated with resistance to these treatments^13,14^. A single antigen targeting CAR T-cell treatment may also be affected by the loss of target as a result of tumor evolution and selection. Targeted antigen-negative relapse is one of the main reasons for resistance to CD19-directed CAR T-cell therapy and accounts for ~9–25% of cases of relapse in other hematological cancers^3,4,6,7^. In addition to antigen loss, immune-mediated rejection of the murine construct may play a role in resistance^15^. We did observe low level anti-drug antibodies (ADAs) at 6 months post first infusion, which persisted during the retreatment. ADA could have potential impact on CAR T cell expansion following second infusion. However, lack of BCMA expression was likely the predominant factor responsible for lack of response to second infusion. The extent of the role of ADA in this setting will need to be ascertained in a larger cohort of patients in the future. Previously, a large study evaluating CD19-targeted CAR T-cell therapy in B-cell malignincies showed that addition of fludarabine to cyclophosphamide-based lymphodepletion before the first infusion and an increased dose in the second infusion compared to the first infusion would increase the response rate^16^, both of which were followed in the currect study.

Here we describe emergence of a clone with loss of BCMA target leading to acquired resistance to retreatment. However, as it is equally important and previously shown in other hematological cancers, there are other possible factors such as microenvironmental changes and immune-mediated rejection of the murine construct that may contribute to resistance to CAR T-cell therapies. Here we only report one mechanism with limited power; however, future studies with larger sample size will be able to determine the dominant resistance factors and expected frequency of each mechanism in MM. We also observed a clonal *TP53* missense mutation (p.P278T) (Fig. 2G) using WES, suggesting that both *TP53* and *BCMA* had deletion in one allele and mutation in the second allele. We analyzed our data from 300 newly diagnosed MM patients and using a conservative estimate observed del16p in 6% patients (44% were subclonal deletions) and, interestingly, it co-occured with del17p in 77% of the del16p patients (sixfold encrichmnet, hypergeometric test *p*-value = 3.38e − 11, Fig. 2I). Importantly, we also observed that 36% of patients with del17p also carried del16p. These results support our previous observation regarding similar relative timing for both deletion events^8,17^, and may highlight the need to carefully examine for *BCMA* gene alterations in patients being retreated with subsequent BCMA targeting therapy at relapse from initial BCMA CAR T-cell treatment. The co-occurrence of 16p deletion in patients with del 17p also underscores the need to further evaluate the role of BCMA targted therapies in high-risk del17p MM. It would also be important to further investigate, with more sensitive methods, the presence and frequency of very low subclonality del16p. In general, WES data from 1300 newly diagnosed MM patients failed to detect any missense or nonsense mutations in BCMA^18^. This suggests that pressure of specific BCMA-targeted treatment can select for a very low level of biallelic deletion (*BCMA and TP53*) in these patients (Fig. 2H). As BCMA has a functional role in MM, such BCMA-independent growth on one hand may indicate a more aggressive phenotype, but it may also suggest a new vulnerability that can be targeted by alternative therapies. Anecdotal instances of post-CAR T-cell sensitivity to various therapies have been reported and the index patient in this report has remained alive 3 years from CAR T-cell therapy.

This case represents molecular characteristics of MM. It identifies significant genomic evolution that may represent clonal selection and/or induction of new changes under the pressure of therapy. Our results suggest that BCMA-negative cell populations may get selected under strong treatments like CAR T-cell therapies. Although the platform we have used was not sensitive enough to detect the presence of low-level resistant cells at an early stage, our results still support a possible role for sensitive and deep sequencing of BCMA locus before CAR T-cell reinfusion or consideration of sequential BCMA-targeted therapies, to identify the outgrowth of a rare MM cell with BCMA loss. Recently, CAR T-cell therapy approach simultaneously targeting dual antigens BCMA and GPRC5D was shown as one approach to prevent BCMA escape-driven relapse^19^. The presence of subclonal changes may also provide clinically important information supporting dual antigen-targeted CAR T cell or other combination or maintenance therapies.

## Methods

### Patient samples

All eight samples for scRNAseq (CD138− sample before the first infusion (S1) and BM mononuclear cells from S2 to S8) and CD138+ sample for WES have been collected from an individual patients’ posterior superior iliac spine area, who was enrolled in a phase I clinical study (CRB-401 ClinicalTrials.gov number, NCT02658929) of bb2121 involving patients with relapsed or refractory MM was initiated. The primary outcome results of this clinical trial have been published^1^. The study was conducted in accordance with the Declaration of Helsinki and International Conference on Harmonisation guidelines for Good Clinical Practice. The protocol was approved by Dana Farber/Harvard Cancer Center Institutional Review Board, and samples and data were obtained after a written informed consent was signed by the patient.

### Single-cell RNA sequencing

For all eight samples, single-cell library constructions were performed using Chromium Single Cell 3′ Reagent kits v2. Each sample was processed individually according to 10× genomics protocols. Poly-A selected transcripts were reverse transcribed and full-length cDNA along with cell barcode identifiers were PCR amplified. The constructed libraries then sequenced with Illumina platform using paired-end sequencing. On average each sample sequenced with 118 M reads (range 87 M–141 M) (Suplementary Table 1). The Cell Ranger Suite (v3.1.0) from 10× genomics with GRCh38 reference genome was used to perform sample de-multiplexing, barcode processing and unique molecular identifier counting. Cellranger mkfastq and count funtions were used to quantify the expression values for captured single cells. The filtered gene-barcode matrix from Cellranger output then used for downstream analysis with Seurat (v3.1.5)^20,21^ to filter out Gel Bead-In Emulsions do not actually contain cells. Estimated number of cells per sample before additional filtering with Seurat^20,21^ was 5864 (range 4868–7801) and mean reads per cell was 20,682 (range 14,730–28,924). Additional quality control measurements can be found in Supplementary Tables 2 and 3.

Filtered counts then transferred to R and Seurat for downstream analysis. Only cells with at least 200 detected features and only feateres that are detected in 3 or more cells were kept for downstream analysis. After these additional filtering steps with Seurat, 4707 cells (range 3075–6818) per sample with 3695 reads per cell were used (Supplemantary Table 1 and Supplementary Fig. 4). Integration of multiple single-cell datasets was performed using anchored Analysis with SCTransform^20,21^ workflow and using 5000 integration features. First 20 dimensions for the Principle Componenet Analysis were used for clustering and Uniform Manifold Approximation and Projection analysis. Single-cell visualizations and downstream marker detections then performed as explained in Seurat website. Resolution was set to 0.3 for the clustering analysis. Known cell-type annotations were perfomed using SingleR(v1.4.0)^22^, as well as known gene surface markers for T, NK, B, plasma cells, monocytes, and erythyrocytes (*CD3D*, *CD3E*, *CD3G*, *CD4*, *CD8A*, *CD5*, *NCAM1*, *CCL5*, *KLRC1*, *KLRD1*, *KLRC2*, *CD79A*, *CD79B*, *CCND1*, *SLAMF7*, *XBP1*, *POU2AF1*, *CD38*, *IRF4*, *CD14*, *FCGR3A*, *CD68*, *PECAM1*, *HBB*) (Supplementary Fig. 1). T-cell subgroups also identified using T-cell subgroup-specific markers (*CD4*, *CD8A*, *CCR4*, *CCR6*, *FOXP3*, *IL2RA*, *CCR7*, *IL7R*, *CD8A*, *CD8B*, *FASLG*, *IFNG*, *NKG7*, *GZMB*, *GZMH*) (Supplemantary Fig. 2). Cytotoxic CD8+ T-cell makers were collected from Zavidij et al.^23^. Copy number analysis for the scRNAseq was done using CONICSmat(v0.1)^24^ and plasma cells were compared with B cells as reference set. Only the chromosomal arms that passed Bayesian information criteria > 0 and adjusted *p*-value < 1e−5 were considered significantly altered. Differentially expressed genes were detected using FindAllMarkers and FindMarkers function in the Seurat^21^ package. Gene-set enrichment analysis was done using molecular signature database (MSigDb) provided by Broad Institute^25,26^.

### Whole exome sequencing

WES data for tumor sample generated from CD138+ cells collected after the second infusion. Peripheral blood mononuclear cells were used as germline control. WES libraries generated using Twist Bioscience Human Core Exome Kit and sequenced as 75 bp paired-end reads with Illumina Novoseq platform. The average sequence coverage for targeted regions was 110× for tumor sample and 602× for germline DNA. We aligned paired-end reads using BWA-mem (v0.7.17-r1188)^27^ to GRCh38. We followed GATK (v4.0.11) best practice to mark duplicated reads with MarkDuplicates function and base quality score recalibration with ApplyBQSR^28^. Mutect2^29^ was used to call mutations. Only mutation calls with at least 10× coverage for tumor and germline samples and passed FilterMutectCalls function were annoted using Variant Effect Predictor from Ensembl (v100). Allele-specific copy number calls as well as ploidy and purity of the sample were analyzed using FACETS (v0.6.1) (Fraction and Allele-Specific Copy Number Estimates from Tumor Sequencing)^30^.

### FISH analysis

CD138+ sorted BM plasma cells were analyzed by FISH using commercially available probes specific for 8q24.1, del13, 17p13.1, gain11, gain1q22, t(4;14), t(11;14), t(14;16), t(14;20) by Mayo Clinic Laboratories. All probes were set up separately and for each probe, plasma cells (if possible) are scored and the result for each probe is reported.

### Other statistical analysis

All other analyses were completed in the R programming language. Data preparation and processing were done using ggplot2, cowplot, and dplyr packages. R maftools was used for downstream analysis for the Single Nucleotide Variant (SNV) and small insertion deletion data. Protein domains were combined with SNV calls using ProteinPaint to generate lollipop plots.

### Detecting CAR+ cells with qPCR

Copies of vector transgene per microgram genomic DNA was determined by quantitative PCR (qPCR) as previously described^1^. Briefly, CD3+ cells were isolated to high purity from whole blood. Genomic DNA from the purified CD3+ cell pellet was extracted and DNA concentration was determined. Purified CD3+ DNA (100 ng) was included in the qPCR reaction for specific quantification of the bb2121 transgene (Psi-gag) and a reference housekeeping gene (*RNaseP*). Detection and quantification of the Psi-Gag sequence and RNaseP were achieved using target-specific oligonucleotide primers and dual-labeled oligonucleotide hydrolysis probes^1^. The amplified targets were detected in real time by Stratagene Mx3005P instrument using TaqMan® Universal PCR Master Mix, no UNG (Thermo Fisher Scientific), and quantified using a standard curve. Quantified copies of vector transgene per reaction is reported as copies per standardized input DNA (100 ng). Primer probe sequences are shown in Supplementary Table 4.

### Reporting summary

Further information on research design is available in the Nature Research Reporting Summary linked to this article.

## Supplementary information

Supplementary Information

Descriptions of Additional Supplementary Files

Supplementary Data 1

Supplementary Data 2

Reporting Summary

## Data Availability

The single-cell RNA sequencing data generated in this study have been deposited in the GEO database under accession code GSE164551. In addition, the single-cell RNA sequencing dataset, BAM file for BCMA locus, SNP counts for allelic copy number, and meta data for single cells after clustering with Seurat are also available in the Harvard Dataverse database under accession code doi:10.7910/DVN/1RKYQ8 [10.7910/DVN/1RKYQ8]. The remaining data are available within the Article, Supplementary Information, or available from the authors upon request.
