# Supplementary material for: Biallelic loss of BCMA as a resistance mechanism to CAR T cell therapy in a patient with multiple myeloma
Source: Nat Commun. 2021 Feb 8;12:868. doi: 10.1038/s41467-021-21177-5 (PMC7870932; doi:10.1038/s41467-021-21177-5)
Supplement: Supplementary file 1 — Supplementary Information [file 41467_2021_21177_MOESM1_ESM.pdf]

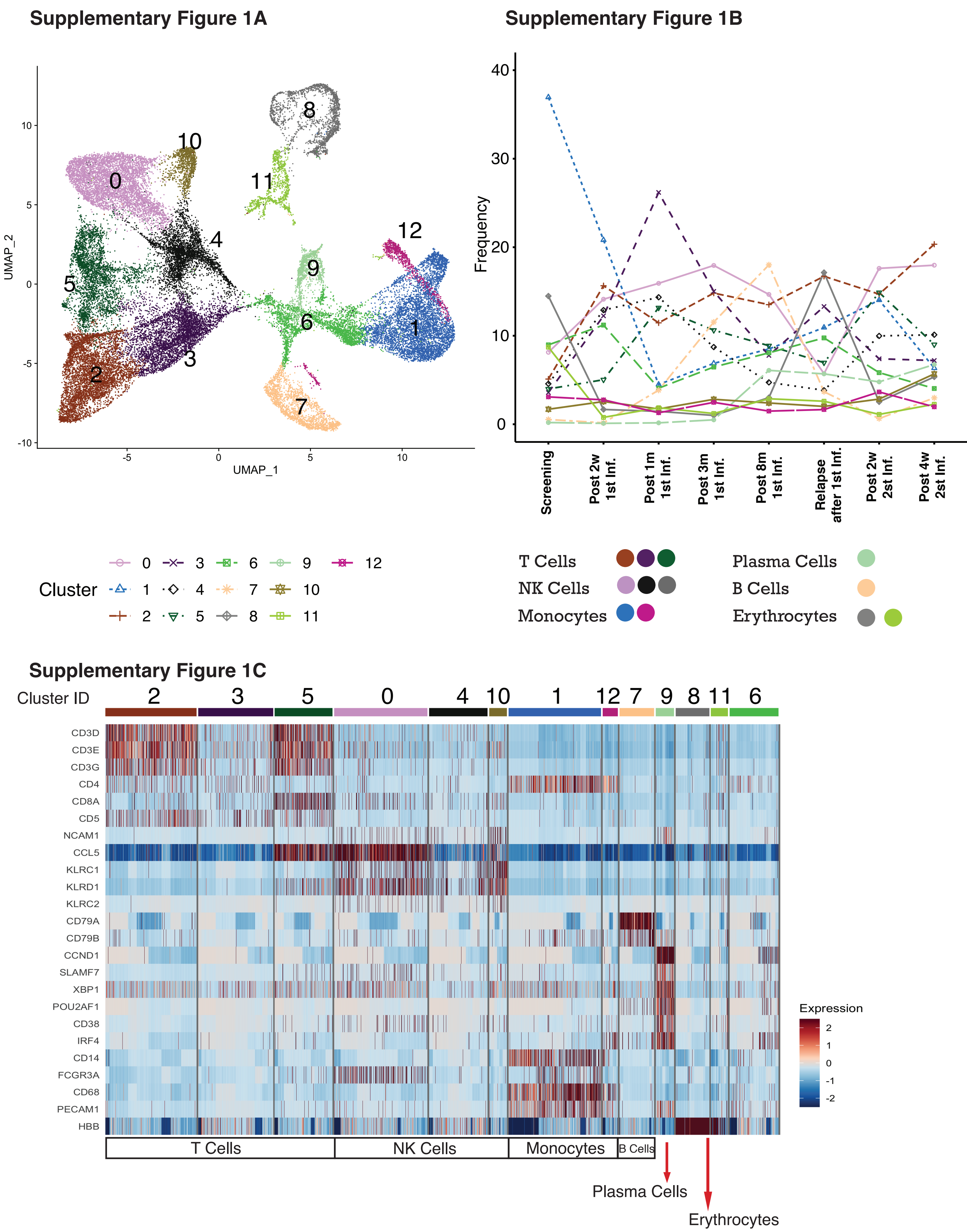


**Supplementary Figure 1:** Clustering analysis of single cell RNA sequencing data. **A)** 13 clusters detected by Seurat. **B)** Percentage of cells (y-axis) in particular cluster (color codes) at each time point (x-axis) evaluated with single cell RNA. **C)** Heatmap of expression values for the marker genes demonstrating cell type identity of clusters. Color codes on the top anotation line refering to clusters in panel A. Columns ordered by cell type identitiy.

## Supplementary Figure 2A

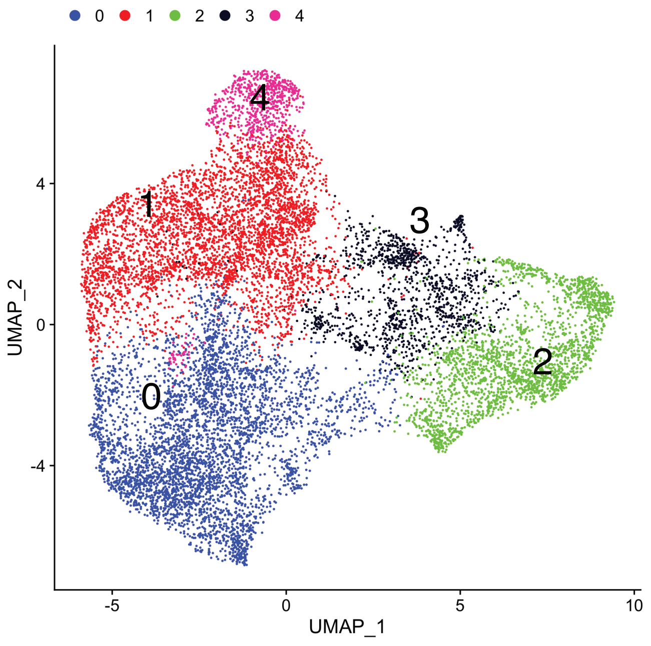


## Supplementary Figure 2B

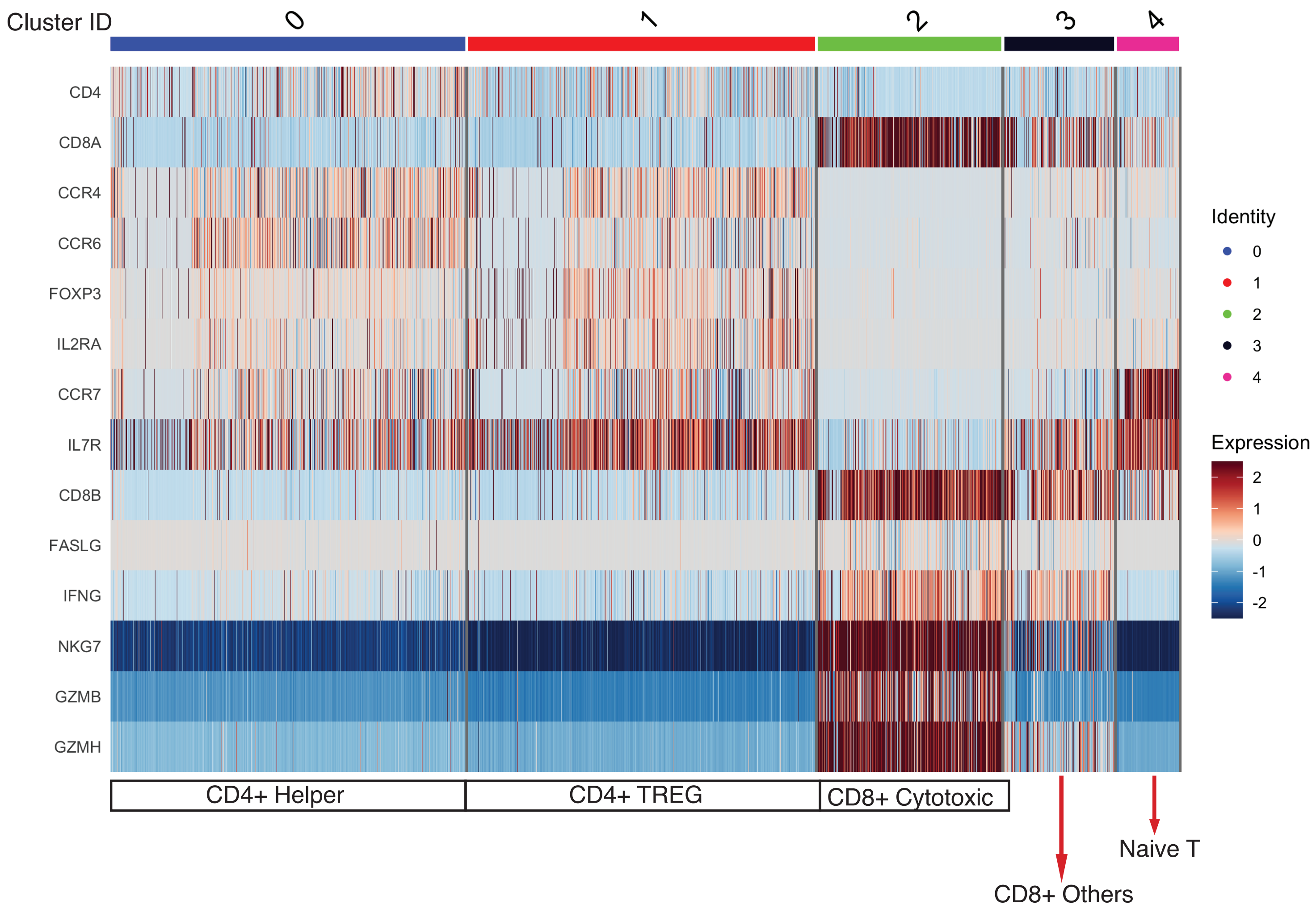


**Supplementary Figure 2:** Clustering analysis of single cell RNA sequencing data for T cells. **A)** 5 clusters detected by Seurat. **B)** Heatmap of expression values for the T cell subset marker genes demonstrating cell type identity of clusters. Color codes on the top anotation line refering to clusters in panel A. Columns ordered by cell type identitiy.

## Supplementary Figure 3A

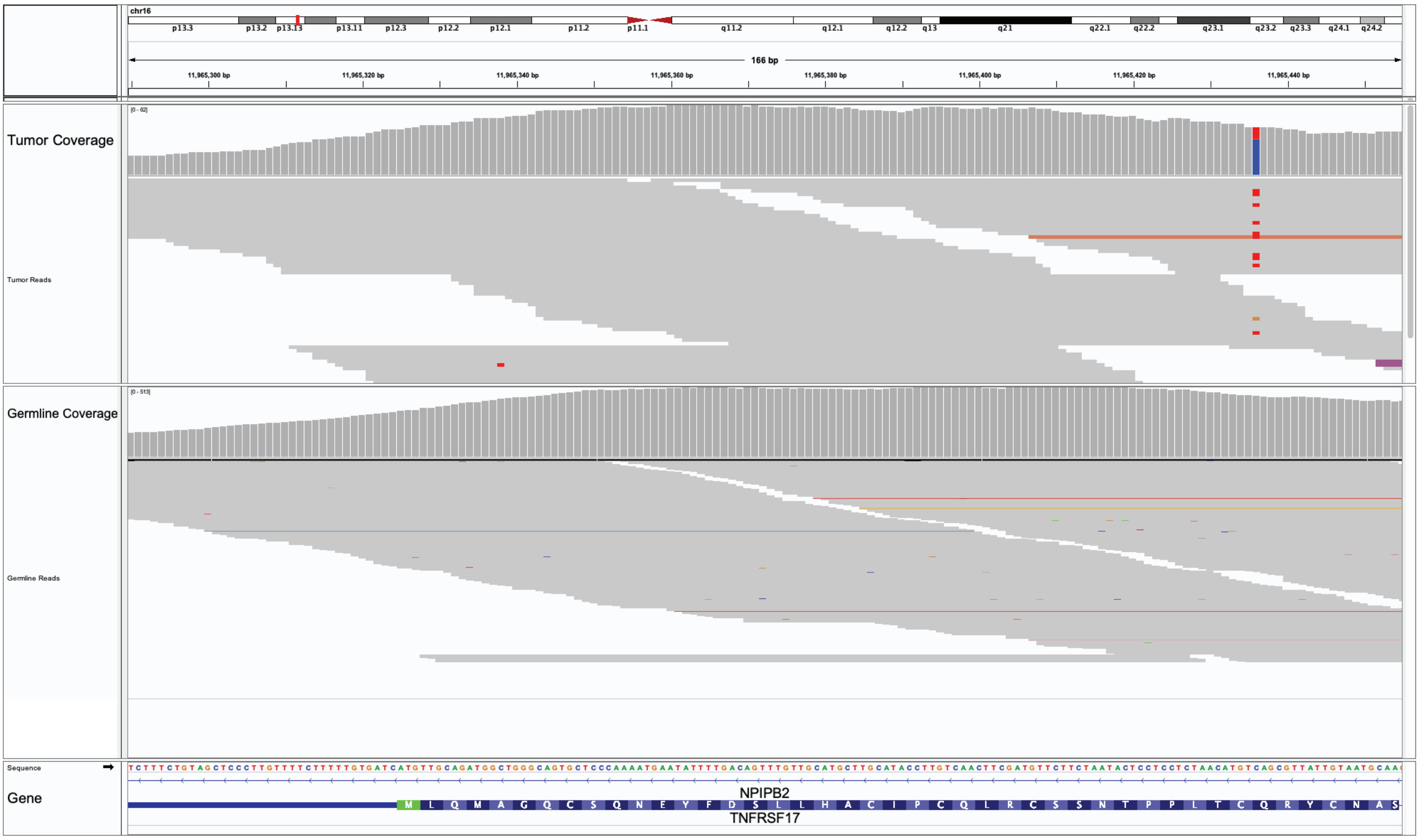


## Supplementary Figure 3B

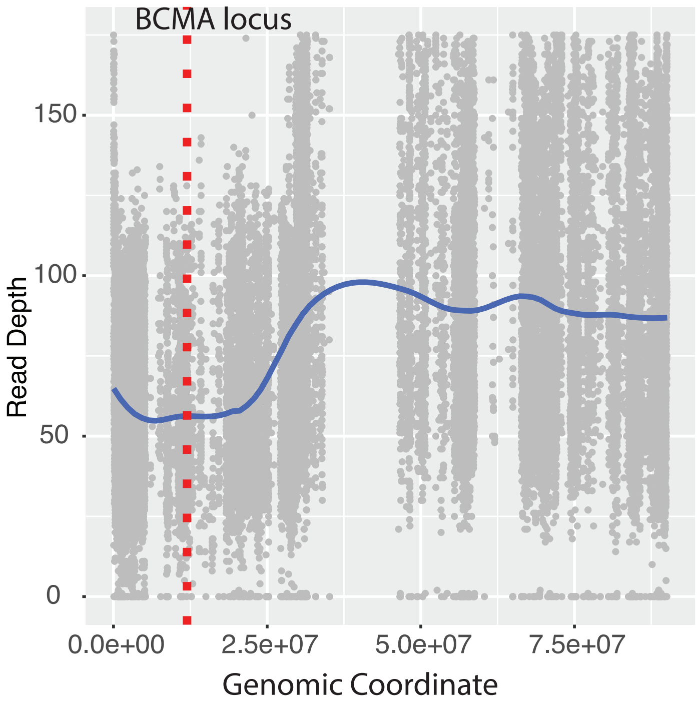


**Supplementary Figure 3:** Coverage data for BCMA deletion and mutation. **A)** Screen shot taken from IGV showing p.Q38* mutation on fist exon of the BCMA gene for MM sample (top panel) and same region in the germline control sample (bottom panel).Blue and red stack bar indicate the number of reads supporting mutated allele (blue) and reference allele (red). **B)** Coverage (read depth, y-axis) for 33489 loci on chromosome 16 shown by genomic coordinates (x-axis) from p arm to q arm. Red vertical dashed line shows BCMA loci.

**Supplementary Figure 4**

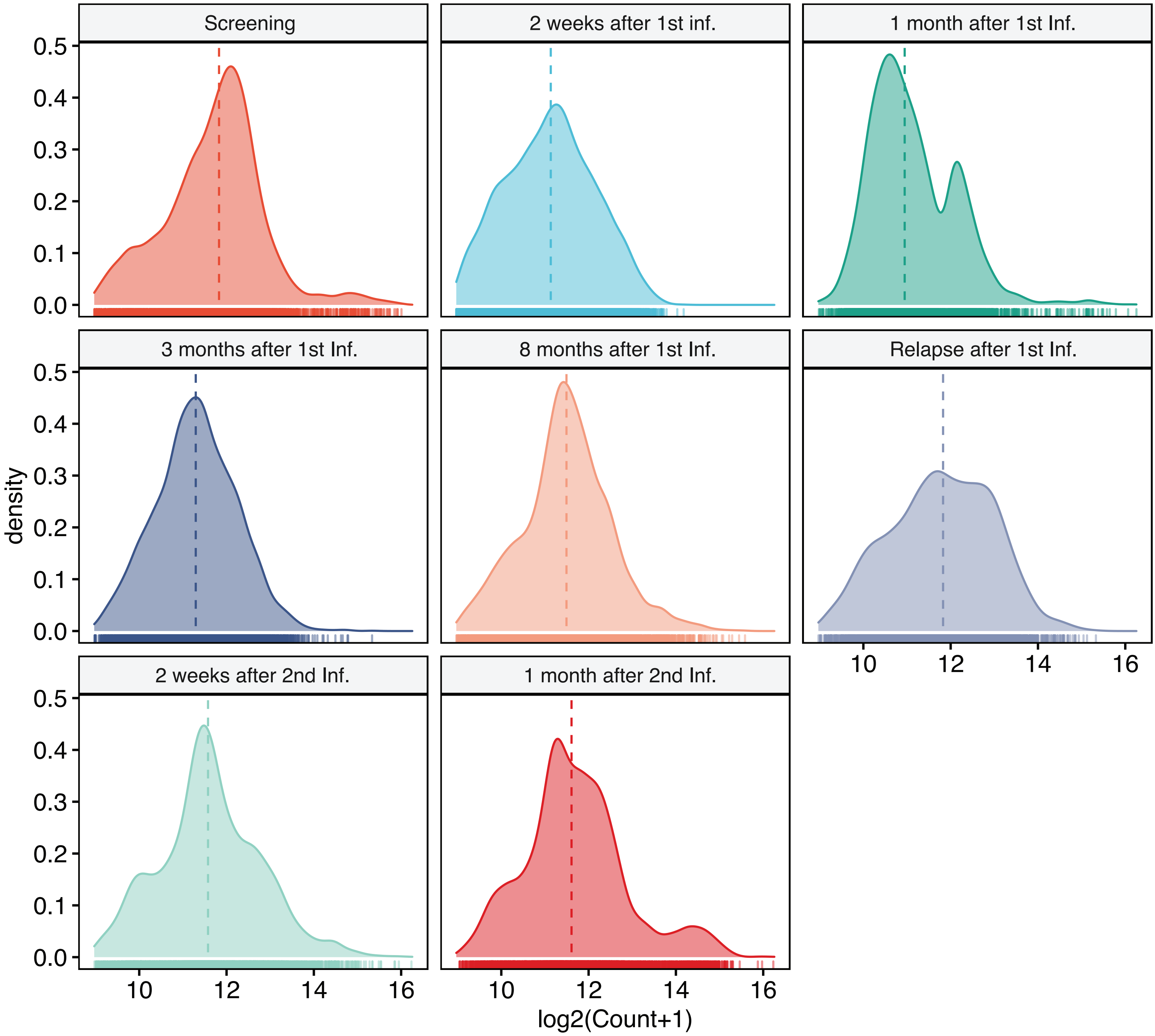


**Supplementary Figure 4:** Distrinution of number of reads per cell after filtering with 10X Genomics CellRanger pipeline and Seurat. Only cells with at least 200 detected features and only feateres that are detected in 3 or more cells were kept for downstream analysis.

## Supplementary Information

**Supplementary Table 1:** Number of cells expression checkpoint inhibitory markers. Cells are counted positive if the normalized expression value is greater than zero. Total number of T cells at each timepoint are shown at the top line (n) and number of cells with expression > 0 at each time point for each CPI maker are given in rows with n (%) format.

| CIM Genes | Screening (n=591) | 2w. After 1st Inf (n=2245) | 1m After 1st Inf (n=2231) | 3m After 1st Inf (n=1865) | 8m After 1st Inf (n=1645) | Relapse (n=1137) | 2w After 2nd Inf (n=1560) | 1m After 2nd Inf (n=1619) |
|---|---|---|---|---|---|---|---|---|
| LAG3 | 300 (50.76%) | 111 (4.94%) | 863 (38.68%) | 606 (32.49%) | 716 (43.53%) | 468 (41.16%) | 811 (51.99%) | 732 (45.21%) |
| PDCD1 | 317 (53.64%) | 39 (1.74%) | 724 (32.45%) | 623 (33.40%) | 790 (43.53%) | 474 (41.69%) | 479 (30.71%) | 832 (51.39%) |
| CD274 | 238 (40.27%) | 17 (0.76%) | 569 (25.50%) | 436 (23.38%) | 492 (43.53%) | 402 (35.36%) | 335 (21.47%) | 683 (42.19%) |
| TIGIT | 317 (53.64%) | 253 (11.27%) | 959 (42.99%) | 687 (36.84%) | 792 (43.53%) | 486 (42.74%) | 580 (37.18%) | 846 (52.25%) |

**Supplementary Table 2:** Quality Control (QC) Metrics after CellRanger pipeline for single cell RNA sequencing

| Samples | Screening - S1 | 2w. After 1st Inf - S2 | 1m after 1st Inf - S3 | 3m after 1st Inf - S4 | 8m after 1st Inf - S5 | Relapse after 1st Inf - S6 | 2w after 2nd Inf -S7 | 1m after 2nd Inf - S8 |
|---|---|---|---|---|---|---|---|---|
| Estimated Number of Cells | 4,868 | 7,801 | 4,903 | 5,191 | 6,559 | 5,167 | 5,999 | 6,420 |
| Mean Reads per Cell | 24,005 | 14,730 | 28,924 | 16,901 | 16,956 | 23,376 | 22,309 | 18,253 |
| Fraction Reads in Cells | 83.30% | 86.10% | 60.80% | 85.80% | 88.00% | 85.20% | 92.80% | 95.00% |
| Median Genes per Cell | 1,045 | 819 | 700 | 888 | 847 | 400 | 883 | 846 |
| Total Genes Detected | 19,254 | 18,879 | 19,061 | 18,528 | 19,456 | 18,435 | 19,233 | 19,147 |
| Number of Reads | 116,856,430 | 114,906,328 | 141,816,230 | 87,733,665 | 111,211,623 | 120,781,352 | 133,829,277 | 117,183,691 |
| Valid Barcodes % | 98.40% | 98.30% | 98.20% | 98.20% | 98.20% | 98.50% | 98.40% | 98.50% |
| Valid UMIs % | 99.90% | 99.90% | 99.90% | 99.90% | 99.90% | 99.90% | 99.90% | 99.90% |
| Median UMI Counts per Cell | 3,537 | 2,208 | 1,902 | 2,426 | 2,696 | 3,540 | 2,759 | 3,311 |

**Supplementary Table 3:** Quality Control (QC) Metrics after Seurat preprocessing for single cell RNA sequencing. Only included cells with >= 200 features detected and features detected in at least 3 or more cells.

| Samples | Screening - S1 | 2w. After 1st Inf - S2 | 1m after 1st Inf - S3 | 3m after 1st Inf - S4 | 8m after 1st Inf - S5 | Relapse after 1st Inf - S6 | 2w after 2nd Inf -S7 | 1m after 2nd Inf - S8 |
|---|---|---|---|---|---|---|---|---|
| Number of Cells After Filter | 4661 | 6818 | 4391 | 4610 | 5454 | 3075 | 4219 | 4430 |
| Mean Reads per Cell | 4709 | 2830 | 3014 | 3105 | 2881 | 3630 | 4470 | 4926 |
| Median Genes per Cell | 1,045 | 819 | 700 | 888 | 847 | 400 | 883 | 846 |

**Supplementary Table 4:** Primers used for qPCR.

| | |
|---|---|
| Psi-Gag forward primer (Sigma or Integrated DNA Technologies) | GGA GCT AGA ACG ATT CGC AGT TA |
| Psi-Gag reverse primer (Sigma or Integrated DNA Technologies) | GGT TGT AGC TGT CCC AGT ATT TGT C |
| Psi-Gag dual-labeled oligonucleotide [robe (5′-FAM, 3′-BHQ-1) (Biosearch Technologies or Integrated DNA Technologies) | FAM/ACA GCC TTC TGA TGT CTC TAA AAG GCC AGG/BHQ-1 |
