# Supplementary material for: Biallelic loss of BCMA as a resistance mechanism to CAR T cell therapy in a patient with multiple myeloma
Source: Nat Commun. 2021 Feb 8;12:868. doi: 10.1038/s41467-021-21177-5 (PMC7870932; doi:10.1038/s41467-021-21177-5)
Supplement: Supplementary file 2 — Descriptions of Additional Supplementary Files [file 41467_2021_21177_MOESM2_ESM.pdf]

Descriptions of Additional Supplementary Files

Supplementary Data 1

Description: Differentially expressed genes between screening and two weeks after first infusion T cells and two weeks after second infusion T cells. Differentially expressed genes were identified with Seurat using Wilcoxon rank sum test and average fold change as well as adjusted p values for multiple testing correction are provided in the table.

Supplementary Data 2

Description: Gene set enrichment analysis results for differentially expressed genes between screening and two weeks after first infusion and two weeks after second infusion (Differentially expressed genes are presented in Supplementary Data 1). p values were calculated usin g h yper geometric distribution and correction for multiple hypothesis testing applied to calculated false discovery rates.
